# New York Pathological Society

**Published:** 1880-06

**Authors:** 


### NEW YORK PATHOLOGICAL SOCIETY.

Stated Meeting April 24, 1880. Dr. Statterthwaite, President, in the Chair.

Dr. Gibney presented a specimen of lupus of the conjunctiva on behalf of a candidate for admission to the society.

Dr. Briddon presented a leg removed by amputation at the knee-joint, from a male mulatto, who suffered from senile gangrene. The line of demarcation had formed half way up the leg. The cause of the gangrene was supposed to be arteritis, but no opportunity had been afforded for a careful examination of the vessels of the part.

He also exhibited a specimen of cancer of the breast, removed by Dr. Harrison, house surgeon of the medical department of the Colored Home.

Dr. Briddon lastly presented a negro child upon whom he had performed Reeve's operation for genu valgum. The history was given as follows :

CASE OF "REEVE'S OPERATION" FOR GENU VALGUM.

Ida Christian, brown-skinned, rachitic child of four years, has been an inmate of the institution for some time. In walking, the feet are separated about eight inches, and the knees alternately cross each other; the deformity is not so marked in the recumbent position.

November 5, 1879, under the influence of ether, and with the spray directed on the parts, an incision, vertical in direction, was made three inches above the internal epicondyle of the right femur, long enough to admit a chisel three-quarters of an inch wide. The instrument was carried down to the bone, and then rotated so that its edge was transversely to the shaft, and was then driven by successive taps of the mallet in an oblique direction, towards the inter-condyloid notch, until it was presumed that it had approached to within a quarter of an inch of the articular surface; the instrument was then withdrawn, and again introduced along the anterior and posterior borders of the first section; the condyle was then detached, with an audible snap, by forcibly straightening the limb, and the wound was protected by the antiseptic dressing.

The same operation was then repeated on the left limb, but before it was possible to detach the condyle on this side, the end of the chisel was driven into the joint, and could be felt underneath the skin; this was also put up with the same precautions as the other, and they were both secured in the straight position by long, padded splints, reaching from the axilla to the side of the foot.

There was scarcely any local or general reaction; the temperature went up one degree the day after the operation, and then subsided to the normal.

Twenty-four hours after the operation the dressings were removed, and found stained with bloody serum; after this there was very littie discharge; the drainage tubes were removed on the sixth day; by the sixteenth day the wounds were healed, and passive motion was commenced and was subsequently used every day, but the side splints were kept applied until the forty-sixth, when the child was put upon her feet; for a couple of days after the attempt of walking, there was a little tumefaction of the joint, and upon its subsidence, passive motion was again used. January 8, 1880, nine weeks after the operation, the limbs are perfectly straight, and the child walks well, but stiffly; passive motion is painful, and resisted by the extensors and by a general effort of the child, but the leg can be flexed to a right angle with the thigh.

I directed the house surgeon that passive motion be done under the influence of ether, so as to forcibly break up any adhesions; two days after, I was informed that even under the anæsthetic the limbs could only be flexed to a right angle, and that on making an effort to increase the range of motion something was felt to snap in the right joint. I saw the child on the following day; found some heat and slight swelling in both joints. I advsed that rest should be enforced for a few days, and that on the subsidence of local disturbance, passive motion should be resumed.

March 22d, I again examined the patient; the limbs were perfectly straight, she could flex them to a right angle, and walked about with ease; on examination, there was complete evidence that there had been separation of the epiphysis of the tibia on the side on which the snap had occurred during the forced flexion under ether, and that union had occurred while the child had been on its feet, and in spite of the passive movements that had been regularly used during the same period; the evidence was a slight anterior angle at the junction of the epiphysis with the shaft, and a bony fusiform swelling, prominent to the eye, but not interfering with the movements of the limb, and I regarded it as a remarkable and interesting feature in the case, that such good results should have obtained under such circumstances.

RECOVERY FROM KNOCK-KNEE.

Dr. Gibney asked if any of the members had seen a case of knock-knee recovery without operation or instrumental aid. Dr. Briddon did not believe such a thing possible in any case of genu valgum, although he had seen recoveries from the ordinary bow-leg.

Dr. Gibney remarked that when the genu valgum depended merely upon relaxation of the internal lateral ligaments of the knee, recovery was possible when suitable support was made, but when the same deformity was due to rachitic enlargement of the internal condyle, a surgical operation was the only trustworthy method of treatment. Dr. Gibney believed in the case of bow-legs said to have recovered, it would always be noticed that the tibia retained its bend. The latter, however, by the growth of the bone, became of comparatively small size, appearing as a slight localized bulging forward and outward just above the ankle.

Dr. Blumenthal stated that he was accustomed, in all cases of bow-legs, to assist nature by supporting the outside of the limbs by a suitable steel splint.

Dr. F. V. White referred to the case of a child who suffered from bow-legs, but who recovered perfectly without mechanical treatment. The patient was nineteen years of age, had perfect limbs, and was somewhat of an athlete.

Dr. Sell exhibited a silver coin (25 cent piece) which was passed per rectum and without treatment three days after it had been swallowed. Dr. White asked if any of the members had seen a case of poisoning from copper after swallowing the ordinary cent, remarking that he had met with one such in his experience.

Dr. Sell presented a small polypus of the cervix removed from a patient aged twenty-nine years, who had suffered from uterine hemorrhages for several years. The growth was removed by the ecraseur and recovery resulted.

A third specimen presented by Dr. Sell consisted of a tænia solium, discharged per rectum from a woman who had been ailing for nine months with the usual symptoms attending the prescence of the worm. The passage of the tænia was effected by a paste formed of pumpkin seed, sugar, and etherial extract of male fern taken fasting.

The Society then went into executive session.

